# Nativity Status Predicts Tobacco Use: A Comparative Analysis of US-Born and Foreign-Born Adults From the Health Information National Trends Survey (HINTS)

**DOI:** 10.7759/cureus.42456

**Published:** 2023-07-25

**Authors:** Valentine C Nriagu, Francis C Okeke, Sandra O Anazor, Nnamdi J Omenuko, Hezborn M Magacha, Chisom M Nwaneki, Tagbo C Nduka

**Affiliations:** 1 Department of Epidemiology and Biostatistics, College of Public Health, East Tennessee State University, Johnson City, USA; 2 Department of Internal Medicine, Maimonides Medical Center, Brooklyn, USA; 3 Department of Medicine, Kharkiv National Medical University, Kharkiv, UKR

**Keywords:** tobacco use, socioeconomic factors, education level, race inequities, racial disparity, tobacco control policy, tobacco cessation, nativity

## Abstract

Introduction

Tobacco smoking remains one of the leading causes of morbidity and mortality globally and in the United States (USA). We hypothesize that US-born naturals have higher odds of tobacco smoking compared to their foreign-born counterparts, and our study aims to assess the relationship between nativity status and odds of tobacco smoking using a nationally representative sample.

Methods

We utilized the Health Information National Trends Survey (HINTS) 5 Cycle 1 (2017) and Cycle 2 (2018) for this study. Our main outcome variable was smoking status divided as ever smoker and never smoker. The main predictor was US birth status. We controlled for sociodemographic characteristics such as age, race, gender, educational status, and marital status. We performed weighted descriptive statistics and bivariate analysis with chi-square for our variables. Unadjusted and adjusted logistic regression was used to ascertain the odds of our outcome given our predictor. Significance was set at 95% confidence, and the alpha level was set to 0.05. All analyses were performed using SAS version 9.4 (SAS Institute Inc., Cary, NC, USA).

Results

Our final sample consisted of 5,677 individuals (weighted: 429,613,693). Of our sample, 36.89% were ever smokers, females were 50.73%, and the majority (57.90%) were high school graduates. In terms of nativity status, those born in the USA were 85.65%, while the non-US-born population was 14.35%.

After adjusting for confounders, we found that non-US-born respondents had 42% lower odds of being ever smokers compared to their US-born counterparts (adjusted odds ratio (AOR) = 0.576; 95% confidence interval (CI) = 0.388-0.854; P = 0.0062). Females were 24% less likely to be ever smokers compared to males (AOR = 0.758; 95% CI = 0.644-0.893; P = 0.0010). Having a bachelor's degree or a graduate degree was associated with 42% and 53% lower odds of being ever smokers compared to high school graduates (AOR = 0.583; 95% CI = 0.474-0.717; P < 0.0001) (AOR = 0.471; 95% CI = 0.377-0.588; P < 0.0001). Whites had 97% higher odds of being ever smokers compared to Hispanics (AOR = 1.977; 95% CI = 1.459-2.679; P < 0.0001).

Conclusion

Our finding of lower odds of tobacco use among foreign-born nationals compared to US-born nationals is consistent with previous studies and suggests the need for equity in tobacco use prevention between the two populations assessed in our study. This is poised to improve overall tobacco use burden, morbidity, and mortality.

## Introduction

Tobacco smoking remains one of the leading causes of morbidity and mortality globally and in the United States (USA) [1,2]. Despite being well known and documented to adversely affect almost every organ and system of the body, over 22.3% of the global population and 19% of the population of the USA continue to use tobacco [3,4]. Before and during the COVID-19 pandemic, former tobacco smokers were found to have higher odds of metabolic conditions compared to never smokers [5]. The cost implication of smoking-related illnesses in the USA has been estimated at over $300 billion yearly with over $225 billion being for direct medical care in adults and over $156 billion going to lost productivity [2,6]. The US Centers for Disease Control and Prevention (CDC) notes that tobacco use causes one in four deaths from cardiovascular disease and nine in 10 cases of lung cancer [7]. Hence, the potential for further increases in chronic disease prevalence and complications with increasing tobacco use is evident.

Most studies have identified factors related to tobacco use among adults globally and characterized them into social factors (norms of the society), environmental factors (advertising), cultural factors (acculturation and history of the tobacco industry in the community), and individual factors [8-11]. However, there is an overwhelming body of evidence on the prevalent role of socioeconomic factors such as low-income levels, social class, lack of academic education, and inequities in tobacco control policies on the continued use of tobacco products [9-11].

In September 2022, there were 47.9 million (approximately 14.9%) foreign-born US residents, the highest ever recorded in US history [12,13]. According to the US official census documentation, a foreign-born individual is anyone who is not a US citizen at birth. This includes those who have become US citizens through naturalization, lawful permanent residents, temporary migrants, humanitarian migrants (refugees and asylees), and unauthorized migrants [14].

Immigration and its effect on the ethnic and racial diversity, culture, demographic size, and characteristics of the US underscore the importance of an in-depth understanding of the diverse characteristics and determinants of health in this important population [13,15]. Recent trends show that 28% of foreign-born naturals do not possess a high school diploma compared to 8% of their US-born counterparts, with the poverty rate being 57% higher among foreign-born naturals who have lived for 20 years in the USA compared to their US-born counterparts [16,17]. These notable differences in sociodemographic and socioeconomic status between these two distinct populations support research in patterns and associations of tobacco use [9].

Although immigrants have been known to have better health outcomes than their US-born counterparts, known as the "Healthy Immigrant Effect," a steady decline in positive health outcomes is well documented [18]. Immigrants who recently migrated to the USA had 52% lower odds of smoking, which decreased to 32% and 18% after 10-15 years and 15 years, respectively, compared to their US-born counterparts [18,19]. The important factors underlying the relationship between immigration status and tobacco use include age at migration, gender, and acculturation [18,20-22]. In the study by Shi et al. among Asian Americans, it was found that foreign-born males who migrated to the USA before age 12 had lower odds of smoking compared to those who migrated in adulthood. This pattern was found to be the opposite in foreign-born Asian females [20]. In a sample of Hispanic young adults in Florida, first-generation immigrants had a lower risk of tobacco and other substance use, which was explained as lower exposure to social stress than foreign-born naturals were exposed to [21]. Living in a neighborhood with a higher foreign-born population was associated with lower risks of substance use with Latino immigrants having a lower likelihood of using tobacco compared to their US-born counterparts [18,23]. A contradictory study showed that children who migrated to the USA before their teen years have higher chances of substance use and psychiatric disorders [18,24]. In a study among Asian American populations in California, females had higher odds of tobacco use compared to males, which some authors have associated with acculturation to the US sociocultural setting [22]. Most evidence shows a pattern of higher odds of tobacco use among US-born natives compared to their foreign-born counterparts.

To our knowledge, no prior studies have used national data to elucidate the relationship between US birth status and tobacco use. Most studies were restricted to various racial groups, hence limiting their external validity. Our study uses nationally representative data to assess this relationship. We hypothesize that US-born naturals have higher odds of tobacco use compared to their foreign-born counterparts, and our study aims to determine how being born in the USA predicts tobacco use.

## Materials and methods

The HINTS 5 Cycle 1 (2017) and HINTS 5 Cycle 2 (2018) dataset is a nationally representative dataset that contains information on adults more than 18 years living in the United States (USA). The HINTS data is collected by the National Cancer Institute using a stratified sampling technique. Questionnaires were mailed to addresses identified by the stratified sampling technique, and an adult identified in the area of residence completes the survey. Inbound telephone calls are provided in both English and Spanish to provide support in completing the questionnaire. Completed questionnaires are mailed back, where they are scanned, verified, cleaned, and edited. Our inclusion criteria included all respondents more than 18 years of age who had completed responses regarding tobacco smoking, age, US nativity status, and race. All respondents who had missing responses to our outcome variables, predictors, and covariates were excluded from this study. Further information on the method of collection of the HINTS data can be found elsewhere [25].

Measures

Our outcome variable was smoking status, which was categorized into two responses: never smoker and ever smoker (we combined respondents who were current and former smokers into this response). Our main predictor was US birth status, which had two categories: yes or no for respondents who were born in the USA and those not born in the USA, respectively. Other predictor variables and confounders we controlled for were race, age group, gender, educational status, and marital status.

The variable race had five categories: White, Black/African American, Hispanic, Asian, and other. The category "other" included American Indian or Alaska Native, Native Hawaiian, or other Pacific Islanders and those with multiple races mentioned. Age was grouped into five categories: 18-34, 35-49, 50-64, 65-74, and 75+. Gender was utilized as is from the questionnaire, which was documented as self-gender and had two categories: male and female.

Educational attainment was categorized into four responses: less than high school, high school graduate, bachelor's degree, and graduate degree. Respondents who had some college were merged with those who were high school graduates. Lastly, we classified marital status into two categories: married and single. Respondents who were married and living as married were categorized as "married," while respondents who were divorced, widowed, separated, single, or never been married were categorized as "single." We accounted for missing variables to prevent distortion of our true association.

Analysis

We performed weighted descriptive statistics of our dependent variables (smoking status), predictors, and confounding variables, and reported percentages and frequency of distributions. We further conducted a weighted bivariate analysis between our outcome variable (tobacco smoking) and our independent variables and covariates (US nativity status, race, age, gender, educational level, and marital status). We utilized the chi-square test in our analysis of the significance of our bivariate analysis as our variables were categorical in nature.

We conducted both weighted unadjusted and adjusted logistic regression models to assess the odds of our outcome given the exposure. Six different simple logistic models were created between our outcome variable and our predictors and covariates. Backward model selection was done to create our final multivariable logistic regression models with predictors having a p-value of <0.2 in our bivariate model included in our final multivariate model. Since marital status had a p-value of >0.2, it was excluded from our multivariate model.

Significance was set at 95% confidence, and the alpha level was set to 0.05. All analyses were performed with SAS version 9.4 (SAS Institute Inc., Cary, NC, USA).

## Results

Descriptive analysis

Our final sample included 5,677 individuals (representing a weighted sample size of about 429,613,693) from both the 2017 and 2018 iterations of our survey after accounting for missing variables. Table 1 shows our final sample size and distribution of our variables. Of this sample, 36.89% were ever smokers and 63.11% were never smokers. The age of respondents showed that those who are 18-34 years made up 24.31%, 35-49 years made up 28.63%, 1,903 (29.69%) respondents were from the age group of 50-64 years, 1,170 (10.42%) respondents were from the age group of 65-74, and those greater than 75 years made up 6.96%. Females made up 50.73% of the respondents, while there were 2,343 (49.27%) males. The majority (57.90%) of the respondents were high school graduates with a bachelor's degree being the second most common highest level of education (20.98%). Other educational qualifications were less than high school in 8.06% and graduate degree in 13.06%. There were 3,118 (55.09%) married respondents and 2,559 (44.91%) single respondents. Those who were born in the USA were 4,880 (85.65%), while non-US-born respondents were 797 (14.35%).

**Table 1:** Representation of all variables in frequencies and percentages of response US: United States

Variable	Frequency (weighted (unweighted))	Percentage
Smoking status		
Ever smoker	158,502,769 (2,179)	36.89%
Never smoker	271,110,924 (3,498)	63.11%
Gender		
Female	217,927,923 (3,334)	50.73%
Male	211,685,770 (2,343)	49.27%
Educational attainment		
Less than high school	34,644,818 (362)	8.06%
High school graduate	248,749,763 (2,679)	57.90%
Bachelor's degree	90,113,130 (1,569)	20.98%
Graduate degree	56,105,981 (1,067)	13.06%
Marital status		
Married	236,692,579 (3,118)	55.09%
Single	192,921,115 (2,559)	44.91%
Age category		
18-34	104,421,171 (734)	24.31%
35-49	122,984,122 (1,203)	28.63%
50-64	127,531,522 (1,903)	29.69%
65-74	44,764,713 (1,170)	10.42%
75+	29,912,164 (667)	6.96%
US birth status		
Non-US born	61,659,601 (797)	14.35%
US born	367,954,092 (4,880)	85.65%
Race		
Asian	22,818,703 (253)	5.31%
Black or African American	44,061,182 (771)	10.26%
Hispanic	66,886,744 (806)	15.57%
Other	11,834,111 (217)	2.75%
White	284,012,952 (3,630)	66.10%

Table 2 shows the results of the test of significance between our predictors and smoking status using chi-square analysis. We observed statistical significance between gender, educational attainment, race, US birth status, age, and our outcome variable, smoking status, respectively. Marital status was not significantly associated with smoking status (P = 0.3881).

**Table 2:** Distribution of predictor variables by smoking status A test of significance with chi-square analysis and corresponding p-values was also included. The significance level is set at <0.05.

Variable	Ever smoker (number (%))	Never smoker (number (%))	p-value
Gender			0.0027
Female	1,170 (46.66%)	2,164 (53.10%)	
Male	1,009 (53.34%)	1,334 (46.90%)	
Educational attainment			<0.0001
Less than high school	159 (10.51%)	203 (6.63%)	
High school graduate	1,218 (65.49%)	1,461 (53.46%)	
Bachelor's degree	485 (15.17%)	1,084 (24.37%)	
Graduate degree	317 (8.83%)	750 (15.53%)	
Marital status			0.3881
Married	1,142 (56.39%)	1,976 (54.34%)	
Single	1,037 (43.61%)	1,522 (45.66%)	
Age category			<0.0001
18-34	175 (13.62%)	559 (30.55%)	
35-49	409 (30.30%)	794 (27.65%)	
50-64	742 (32.99%)	1,161 (27.75%)	
65-74	552 (13.89%)	618 (8.39%)	
75+	301 (9.18%)	366 (5.67%)	
US birth status			<0.0001
Non-US born	193 (8.42%)	604 (17.82%)	
US born	1,986 (91.58%)	2,894 (82.18%)	
Race			<0.0001
Asian	52 (2.66%)	201 (6.86%)	
Black or African American	266 (8.97%)	505 (11.01%)	
Hispanic	220 (10.43%)	586 (18.57%)	
Other	94 (3.36%)	123 (2.40%)	
White	1,547 (74.58%)	2,083 (61.16%)	

Predictors of smoking status

We conducted weighted bivariate and multivariate logistic regression to assess the odds of smoking given our main predictor variables and associated sociodemographic characteristics. The results of these models are given in Table 3.

**Table 3:** Simple and multivariate logistic regression for each predictor variable with corresponding odds ratio, confidence interval, and p-value CI: confidence interval

Variable	Unadjusted			Adjusted		
	Odds ratio	95% CI	p-value	Odds ratio	95% CI	p-value
Gender						
Male	Reference	Reference	Reference	Reference	Reference	Reference
Female	0.772	0.653-0.913	0.0027	0.758	0.644-0.893	0.0010
Educational attainment						
High school graduate	Reference	Reference	Reference	Reference	Reference	Reference
Less than high school	1.294	0.884-1.893	0.184	1.663	1.100-2.515	0.0161
Bachelor's degree	0.508	0.416-0.621	<0.0001	0.583	0.474-0.717	<0.0001
Graduate degree	0.464	0.372-0.578	<0.0001	0.471	0.377-0.588	<0.0001
Age category						
18-34	Reference	Reference	Reference	Reference	Reference	Reference
35-49	2.458	1.760-3.432	<0.0001	2.592	1.798-3.738	<0.0001
50-64	2.667	1.950-3.647	<0.0001	2.516	1.792-3.533	<0.0001
65-74	3.716	2.721-5.075	<0.0001	3.322	2.336-4.723	<0.0001
75+	3.630	2.535-5.200	<0.0001	3.024	2.032-4.500	<0.0001
US birth status						
US born	Reference	Reference	Reference	Reference	Reference	Reference
Non-US born	0.424	0.321-0.560	<0.0001	0.576	0.388-0.854	0.0062
Race						
Hispanic	Reference	Reference	Reference	Reference	Reference	Reference
Asian	0.689	0.408-1.162	0.1621	1.250	0.717-2.179	0.4289
Black or African American	1.452	0.998-2.113	0.0515	1.241	0.862-1.785	0.2439
Other	2.486	1.464-4.221	0.0008	2.705	1.542-4.747	0.0006
White	2.171	1.653-2.851	<0.0001	1.977	1.459-2.679	<0.0001

In our univariate models, we found that compared to US-born individuals, non-US-born individuals had about 58% lower odds of being ever smokers (odds ratio (OR) = 0.424; 95% confidence interval (CI) = 0.321-0.560; P < 0.0001). Respondents with bachelor's degrees and graduate degrees had about 50% and 54% lower odds of being ever smokers when compared to high school graduates (OR = 0.508; 95% CI = 0.416-0.621; P < 0.0001) (OR = 0.464; 95% CI = 0.372-0.578; P < 0.0001).

Age showed a characteristic increase in odds with every increase in the age category. When compared to those between 18 and 34 years, those between 35 and 49 years had 2.45 times higher odds of being ever smokers (OR = 2.458; 95% CI = 1.760-3.432; P < 0.0001), those between 50 and 64 years had 2.67 times higher odds (OR = 2.667; 95% CI = 1.950-3.647; P < 0.0001), those between 65 and 74 years had 3.72 times higher odds of being ever smokers (OR = 3.716; 95% CI = 2.721-5.075; P < 0.0001), and those more than 75 years had 3.63 times higher odds (OR = 3.630; 95% CI = 2.535-5.200; P < 0.0001). When we analyzed the effect of race, we found that only Whites and other races were significantly associated with smoking status compared to Hispanics. Whites had 2.17 times higher odds of being ever smokers, and other races had 2.486 higher odds of being ever smokers compared to Hispanics (OR = 2.171; 95% CI = 1.653-2.851; P < 0.0001) (OR = 2.486; 95% CI = 1.464-4.221; P = 0.0008).

After model selection, our final multivariate logistic model showed that compared to US-born respondents, non-US-born respondents had 42% lower odds of being ever smokers (AOR = 0.576; 95% CI = 0.388-0.854; P = 0.0062). Gender was significantly associated with smoking status as females had 24% lower odds of being ever smokers compared to males (AOR = 0.758; 95% CI = 0.644-0.893; P = 0.0010).

While respondents with less than high school educational attainment had 66% higher odds of being ever smokers compared to high school graduates (AOR = 1.663; 95% CI = 1.100-2.515; P = 0.0161), respondents with bachelor's and graduate degrees had 42% and 53% lower odds of being ever smokers compared to high school graduates, respectively (AOR = 0.583; 95% CI = 0.474-0.717; P < 0.0001) (AOR = 0.471; 95% CI = 0.377-0.588; P < 0.0001).

All our age categories had higher odds of being ever smokers when compared to those between 18 and 34 years. Respondents between 35 and 49 years had 2.59 times higher odds (AOR = 2.592; 95% CI = 1.798-3.738; P < 0.0001), those between 50 and 64 years had 2.51 times higher odds of being ever smokers (AOR = 2.516; 95% CI = 1.792-3.533; P < 0.0001), respondents between 65 and 74 years had 3.32 times higher odds of being ever smokers (AOR = 3.32; 95% CI = 2.336-4.723; P < 0.0001), and those more than 75 years had 3.02 times higher odds compared to respondents in the 18-34 years category (AOR = 3.024; 95% CI = 2.032-4.500; P < 0.0001).

Like our univariate model, when the relationship of race was assessed, only Whites and other races had a significant association with being ever smokers when compared to Hispanics. Whites had 97% higher odds of being ever smokers, and other races had 2.71 times higher odds of being ever smokers compared to Hispanics (AOR = 1.977; 95% CI = 1.459-2.679; P < 0.0001) (AOR = 2.705; 95% CI = 1.542-4.747; P = 0.0006).

## Discussion

The main finding of assessing two years of nationally representative data shows that smoking status was related to nativity status, age, gender, educational status, and race. Our results showed that the population of foreign-born nationals was 14.35%. This is similar to the national estimate as of September 2022, which was about 14.9% [12,13]. This increases our study's validity and further improves external validity in the public health sphere of today.

Previous studies have shown that foreign-born nationals have lower odds of tobacco smoking [18-22]. This is similar to our findings that found 42% lower odds of tobacco smoking among foreign-born nationals compared to US-born nationals. These studies consistently show a range of lower odds between 32% and 52%, with the odds decreasing by the number of years lived in the USA [18,19].

Assessing the effect of age on tobacco smoking status, we found that the odds of being ever smokers of tobacco were higher as the age of respondents increased. Those who were more than 35 years had 2-3 times higher odds of being ever smokers compared to those less than 35 years. Based on the literature, age at migration plays an important role in influencing tobacco use, with those who migrated before age 12 having lower odds of being ever smokers compared to those who migrated as adults. However, since our study assessed the effect of age on the overall population of ever smokers of tobacco, we could not ascertain the effect of age at migration on the risks of being ever users of tobacco. Part of the challenges included the small sample of foreign-born nationals, which precluded further sub-stratified analysis.

The literature has consistently shown lower odds of tobacco use among females compared to males [26,27]. This was consistent with our study, which found 24% lower odds of being ever smokers in females compared to males. Similarly, the lower the educational attainment, the higher the odds of being ever smokers of tobacco [26,28,29]. This was consistent in our study where we found that compared to high school graduates, those with less than high school education had higher odds of being ever smokers, while those with bachelor's and graduate degrees had lower odds of being ever smokers. This could be explained by the constant exposure to anti-tobacco advertisements and the professional status that is associated with higher educational qualifications.

Our study failed to sub-stratify these findings between both foreign-born and US-born nationals to compare the findings. The challenges faced in this were due to the small sample size of foreign-born nationals in our study population.

Strengths

Our study has some strengths that ensure its increased internal and external validity. Firstly, we utilized a national sample in our analysis, which improves the overall estimate between both populations we compared. Also, our dataset utilized weighting in the data collection and survey, thereby increasing the generalizability and estimate of the results to the general population. By combining two iterations of the dataset, we had a larger sample size, which increased our power to detect true associations between our outcome and predictor variables.

Finally, our study found a similar direction of risk compared to previous studies and further adds to the body of evidence on that.

Limitations

Our study had a small population of foreign-born individuals compared to US-born nationals, and this may affect the true outcome of the association due to the skewness of the data. Similarly, due to the small sample size of foreign-born nationals, we could not do a sub-stratified analysis of the odds of our outcome by country of origin and by years of living in the USA.

Recommendations and public health implications

Our study adds to the body of evidence on the predictors of tobacco use, with US-born nationals having higher odds compared to their foreign-born counterparts. This underscores the need for proper studying of the sociocultural factors and tobacco control policies of foreign countries for effective adaptation in the US tobacco control sphere. This is poised to help decrease tobacco-related costs of care, morbidity, and mortality in the USA.

Also, as the immigrant population continues to increase, the need for equity in lowering tobacco use should be of importance, as US-born nationals are more likely to be affected by the burden.

We further recommend future studies to perform pooled analysis of studies and conduct sub-stratified analysis to detect the association by country of birth and years of living in the USA.

## Conclusions

In conclusion, our data and study showed that foreign-born nationals living in the USA have lower odds of tobacco smoking compared to US-born nationals. Other sociodemographic factors such as age, gender, educational status, and race also have a role to play in individual risk of tobacco use. We recommend effective public health policies and tobacco prevention strategies targeted at improving equity in tobacco use cessation campaigns. Modeling of tobacco control and sociocultural factors of foreign nations and its application in the US sphere may balance out the disparities as detailed in our study.
